# Endochondral Ossification Is Accelerated in Cholinesterase-Deficient Mice and in Avian Mesenchymal Micromass Cultures

**DOI:** 10.1371/journal.pone.0170252

**Published:** 2017-01-24

**Authors:** Janine Spieker, Thomas Mudersbach, Astrid Vogel-Höpker, Paul G. Layer

**Affiliations:** Developmental Biology and Neurogenetics, Technische Universität Darmstadt, Schnittspahnstrasse 13, Darmstadt, Germany; Weizmann Institute of Science, ISRAEL

## Abstract

Most components of the cholinergic system are detected in skeletogenic cell types *in vitro*, yet the function of this system in skeletogenesis remains unclear. Here, we analyzed endochondral ossification in mutant murine fetuses, in which genes of the rate-limiting cholinergic enzymes acetyl- (AChE), or butyrylcholinesterase (BChE), or both were deleted (called here A^-^B^+^, A^+^B^-^, A^-^B^-^, respectively). In all mutant embryos bone growth and cartilage remodeling into mineralizing bone were accelerated, as revealed by Alcian blue (A-blu) and Alizarin red (A-red) staining. In A^+^B^-^ and A^-^B^-^ onset of mineralization was observed before E13.5, about 2 days earlier than in wild type and A^-^B^+^ mice. In all mutants between E18.5 to birth A-blu staining disappeared from epiphyses prematurely. Instead, A-blu^+^ cells were dislocated into diaphyses, most pronounced so in A^-^B^-^ mutants, indicating additive effects of both missing ChEs in A^-^B^-^ mutant mice. The remodeling effects were supported by *in situ* hybridization (ISH) experiments performed on cryosections from A^-^B^-^ mice, in which Ihh, Runx2, MMP-13, ALP, Col-II and Col-X were considerably decreased, or had disappeared between E18.5 and P0. With a second approach, we applied an improved *in vitro* micromass model from chicken limb buds that allowed histological distinction between areas of cartilage, apoptosis and mineralization. When treated with the AChE inhibitor BW284c51, or with nicotine, there was decrease in cartilage and accelerated mineralization, suggesting that these effects were mediated through nicotinic receptors (α7-nAChR). We conclude that due to absence of either one or both cholinesterases in KO mice, or inhibition of AChE in chicken micromass cultures, there is increase in cholinergic signalling, which leads to increased chondroblast production and premature mineralization, at the expense of incomplete chondrogenic differentiation. This emphasizes the importance of cholinergic signalling in cartilage and bone formation.

## Introduction

Endochondral ossification in vertebrates, that is formation of long bones, presents an excellent model to analyze cellular and molecular functioning of so-called non-neuronal cholinergic systems (NNCS). The relevance of this for human health and disease has recently gained increasing attention [1]. For example, the neurotransmitter acetylcholine (ACh) itself, or activation of nicotinic and muscarinic receptors have proliferative and anti-apoptotic effects in many cell types [2, 3]. In bony tissues, all components of the cholinergic system are widely expressed, including ACh, the ACh-synthesizing enzyme choline acetyltransferase (ChAT), ACh receptors (AChRs) and ACh-degrading enzymes, acetyl- (AChE) and butyrylcholinesterase (BChE) [4–7]. AChE expression has been identified in the condensing mesenchyme, localized in pre-cartilage cell clusters during chick and rat limb development [8–10], and also in differentiating osteoblasts [11, 12]. Functioning of cholinesterases in developmental processes deserves particular attention, since—due to their high turnover rate—they represent rate-limiting components within classical cholinergic signaling. Indeed, using bead transplantations including cholinergic components into chicken limb buds, skeletogenesis was clearly accelerated by cholinergic stimulation [13]. Moreover, both ChEs have been associated with non-neuronal functions. For example, BChE could have a role in cell proliferation during embryonic development and in cancers [7, 10]. AChE, on the other hand, exerts additional enzymatic and/or structural functions, including those involved in cellular adhesion [14–17]. Further strong support for AChE´s particular role in bone formation is based on the fact that the skeletogenic master regulator Runx2 binds to the AChE promoter [18].

The process of *endochondral ossification* typifies skeletogenesis of long bones, ribs and vertebrae, whereby a cartilage that will eventually become bone, is replaced by calcified tissue (note: major parts of the skull follows "direct ossification", which is not dealt with here). This process is initiated by distinct steps of chondrocyte differentiation [19, 20], and the entire process is regulated by a network of skeletogenic master genes [21, 22]. As mesenchymal stem cells proliferate and differentiate into chondrocytes, the cells begin to produce an extracellular matrix (ECM), rich in type II collagen (Col-2). In this process, highly proliferative chondrocytes become arranged in columns and differentiate into pre-hypertrophic and hypertrophic chondrocytes, a step associated with exchanging Col-2 by Col-10. To promote further chondrocyte differentiation, pre-hypertrophic chondrocytes transiently express Indian hedgehog (*Ihh*) signaling [23, 24]. Cell division only ceases when chondrocytes differentiate into hypertrophic chondrocytes, a step dependent on Ihh and sustained by Runx2 expression. Specific types of AChRs have been detected in resting, proliferating (*pc*) and pre-hypertrophic chondrocytes (*ph*) of the murine growth plate (GP), as well as in osteoblasts, spongy bone and periosteum, but not in hypertrophic chondrocytes (*hc*) [4, 25–27]. Terminal hypertrophic differentiation is accompanied by a massive increase in cell volume and expression of alkaline phosphatase (*ALP*), an enzyme required for mineralization of cartilage [20], which is the first step in *cartilage remodeling*. Only terminally differentiated hypertrophic chondrocytes express the cartilage-degrading matrix-metalloproteinase 13 (*Mmp-13*) [28]. The remaining matrix from degraded hypertrophic chondrocytes serves as scaffold for mineral deposition during ossification. Runx2^+^ and Col-2^+^ osteoblastic progenitors located in the perichondrium will differentiate into osteoblasts and become translocated into the future bone marrow cavity, before and during vascularization [21, 22, 29–32].

Research on cholinergic regulation of bone metabolism has been mainly focused on its involvement in diseases and pathological conditions [2, 5, 6, 27]. Thus, many studies have demonstrated that smoking during pregnancy, e.g. the ACh agonist nicotine affected bone mass and body weight in childhood, and also exerted negative health effects on bones in adulthood [2, 33, 34]. In fact, exposure to organophosphates and carbamates (pesticides, insecticides, environmentally disrupting agents, nerve gases, cholinergic therapeutics [35]), skeletal genetics (e.g., achondroplasia, dwarfism; [36–38]), and even skeletal ageing in elderly people (osteoporosis, arthritis [39–41]) could depend on cholinergic regulation. Here we investigated the effects of deletion or inhibition of ChEs affects on bone formation in embryonic mouse and chick models. We analyzed three ChE mutant mice, in which either one, or both ChEs were deleted. In all cholinesterase mutants from E13.5 until birth, growth dynamics and endochondral ossification were much affected, along with disruption in expression of skeletogenic master genes and a remarkable loss of proteoglycans. Notably, all ChE mutants presented distinct phenotypic differences, indicating that both ChEs contribute differently to the entire process of skeletogenesis. Independently, an improved *in vitro* micromass culture system of chicken limb bud cells showed that cholinergic stimulation, or AChE inhibition accelerated chondrogenic differentiation and mineralization. In summary, this study demonstrates leading roles of AChE and BChE in bone development, predominantly but not exclusively by terminating ACh-mediated activation of nAChR. The biomedical implications of these findings are briefly discussed.

## Materials and Methods

### Animals

Formalin-fixed samples of WT, AChE^-/-^/BChE^+/+^, AChE^+/+^/BChE^-/-^, and AChE^-/-^/BChE^-/-^ knockout mouse embryos were kindly provided by Dr. O. Lockridge (University of Nebraska Medical Center, Omaha, USA). The AChE^-/-^ mice were generated in the 129SV strain and characterized as described previously [42]. After birth, double knockout mice (AChE^-/-^/BChE^-/-^) did not breathe and hence died immediately after birth. AChE^-/-^ KO mice survive to adulthood when maintained on a liquid diet [43], however, these mice do not breed. Therefore, the AChE knockout colony was maintained by breeding heterozygotes. BChE^-/-^ knockout mice were produced by gene targeting and bred to mice in strain 129S1/SvImJ [44]. BChE^-/-^ mice are healthy and indistinguishable from wild-type mice under normal conditions. The AChE^-/-^/BChE^-/-^ phenotype was analyzed and the mice were genotyped by PCR. Samples were immediately taken for genotyping and then mice were fixed in 4% PFA/PBS, pH 7.4.

**Ethics statement:** 1. For studies on mice: all tissue materials from mice as used in this study were provided as fixed samples from Dr. O. Lockridge (Omaha, USA; for ethics declarations see [36–38]), except for *in situ* hybridisation results of Runx2 and Ihh, which were derived from mutant KO mice from the same lines, raised in our own colony at TU Darmstadt (permission, Regierungspräsidium Giessen, AZ IV 44-53r30.03TUD10.01). 2. For studies on chick embryos: according to German animal welfare regulations ("Deutsches Tierschutzgesetz"), chicken embryos before hatching are not assigned the legal status of "animals"; therefore approval from ethics committee was not required for this study.

### Chick limb bud micromass culture system

#### Media

The culture medium consisted of a commercial Dulbecco’s modified Eagle’s medium supplemented with 10% fetal calf serum, 2% fractionated chick embryo extract, 1% L-glutamine (all from Gibco, Berlin, Germany), penicillin (20μg/ml), streptomycin (20μg/ml), and gentamycin (20μg/ml). The differentiation medium consisted of Dulbecco’s modified Eagle’s medium supplemented with 10% fetal calf serum, 2% fractionated chick embryo extract, ascorbic acid (20μg/ml), NaHCO_3_ (3,7mg/ml), glucose (1g/l), CaCl_2_ (1mM), KH_2_PO_4_ (3mM) and 1% L-glutamine (all from Gibco, Berlin, Germany), penicillin (20μg/ml), streptomycin (20μg/ml), and gentamycin (20μg/ml).

#### Procedure

Except for minor changes, the micromass culture was performed as described previously [45, 46]. Chicken embryos of HH stage 22–24 were used to cultivate mesenchymal cells of the chick wing buds. The wing buds were dissociated by enzymatic treatment with 0.1% trypsin (Worthington Biochemicals/Cell Systems, Remagen, Germany) and 2.4U of dispase II (Sigma Aldrich, St. Louis, MO) for 10 min at 37°C under continual shaking. The loosened ectoderm was then removed manually with fine preparation forceps. Purity of the mesenchymal tissue (absence of epithelial cells) was established microscopically. The mesenchymal tissue was mechanically dissociated in culture medium, and the cell number was adjusted to 2x10^7^ cells/ml. A 10μl drop, containing 2x10^5^ cells, was carefully placed in the center of each cavity of a 24-well plate. The cell drops were incubated for 1 h at 37°C and 5% CO_2_; subsequently 500μl aliquot of culture medium was added to each cavity. After two days of incubation, the culture medium was changed and replaced with 1 ml of fresh differentiation medium and afterwards changed every two days. To study the cholinergic system *in vitro*, the micromass cultures were treated with different concentrations (conc., see in Results and Figure legends) and combinations of either BW284c51 (specific AChE inhibitor), nicotine (an ACh agonist) or MLA (an antagonist to α7-nAChR), all 3 purchased from Sigma.

### Histological stainings of micromass cultures

The cultures were washed with PBS and fixed with 4% PFA for 2 h. After removal of PFA, the cultures were stained by Alcian Blue (A-blu), Alizarin Red (A-red), or Alkaline phosphatase (ALP). For Alcian Blue staining, the cultures were pre-incubated with 3% acetic acid for 10 min, then incubated in Alcian Blue solution (0.1mg/ml) for 1hr at room temperature. The cultures were then washed with PBS and immersed in glycerol. Alizarin Red staining was performed by pre-incubating the cultures in 0.5% KOH for 10 min, and then incubated in 0.1% A-red staining solution for 1 hr at room temperature. The coloring reaction was stopped by washing with PBS, and then cultures were immersed in glycerol. Alkaline phosphatase, which catalyzes the mineralization of bone, was studied *in vitro*. The cultures were pre-incubated twice in freshly prepared alkaline phosphatase buffer (100mM Tris, 50mM MgCl_2_, 100mM NaCl, 0.1% Tween 20 in distilled water), and then treated with staining solution (3.5 μl BCIP + 4.5 μl NBT/ml alkaline phosphatase buffer) for 1 hour in dark. The reaction was stopped by washing the cultures in PBS, and finally immersed in glycerol.

### Skeletal preparation and histology

For morphological analysis embryos were eviscerated and the skin was removed. After overnight fixation in 4% PFA at 4°C, embryos were stained in Alcian blue solution (0.1mg Alcian blue in 2% acetic acid/EtOH) overnight. After several hours in 95%, 70%, 40% and 15% ethanol, they were transferred to a 1% trypsin solution for 2 hours to digest and clear the tissue. After staining in Alizarin red S solution for 16 h (40mg/l Alizarin red in 0.5% KOH), skeletons were cleared in a series of 0.5% KOH/25% glycerol, 0.5% KOH/50% glycerol and stored in 0.5% KOH/70% glycerol at 4°C.

For histochemistry, limbs (whole front, or hind limbs) from newborn mice were fixed in 4% PFA in phosphate-buffered saline at 4°C overnight, and were dehydrated in 30% sucrose solution for 2 days. Sagittal 12 μm cryosections were prepared, stained with Alcian blue solution (see above) and counterstained with 0.1% solution of nuclear fast red to assess histology of limb growth plates.

### Cholinesterase stainings

Acetylcholinesterase staining was performed using the Karnovsky and Roots technique [47]. In brief, frozen cryosections were dried on a heating plate, incubated twice for 30 min in Tris-Maleate buffer (0.1M, pH 6.0) and treated with the reaction buffer (0.1M Tris-Maleate buffer, 0.1M sodium citrate, 30 mM copper sulfate and 5mM potassium hexacyanoferrate). The reaction was conducted for 1 hour at 37°C until appropriate staining was reached, with 6 mM acetylthiocholine iodide (0.72mg/ml) as substrate and 0.1mM iso-OMPA to inhibit BChE, which led to the production of a brown insoluble precipitate (Hatchett’s brown) at the site of enzymatic AChE activity, which was inspected under the light microscope. For visualizing BChE activity, butyrylthiocholine iodide (1mg/ml) was used as substrate, and 0.5mM BW284c51 to inhibit AChE, respectively.

### Staining of alkaline phosphatase (ALP) activity

ALP activity was examined on cryosections of embryonic limbs by staining with a BCIP/NBT solution (4.5μg/ml NBT+3.5μg/ml BCIP in DMF) in alkaline phosphatase buffer, consisting of 100mM Tris (pH 9.5), 50mM MgCl_2_, 100mM NaCl, 0.1% Triton X-100, 0.1% Tween-20.

### In situ hybridization (ISH)

*In situ* hybridization (ISH) on mouse femur cryosections with digoxigenin-labeled mouse antisense RNA probes was performed, as described previously [48]. Briefly, newborn mouse hind limbs were fixed in 4% paraformaldehyde in PBS overnight and dehydrated in 30% sucrose solution for cryosectioning. Riboprobes for *Col II* [43], *Col X* [44], *Ihh* [45], *Mmp13* [46], and Runx2 were kindly provided by Dr. Vortkamp (University of Essen, Germany).

### Statistics

For descriptions of A-blu and A-red-stained whole mounts of mice embryos, 20 AChE^-/-^/BChE^-/-^ double knockout mice and 10 wild type mice of stages E13.5 to P0 were used (all of them were shipped as fixed samples from the Lockridge lab). For histochemical analyses (IHC, ISH), limbs from 20 AChE^-/-^/BChE^-/-^ double knockout mice and 14 wild type mice of stages E13.5 to P0 were used (all of them were shipped from Omaha (as above), except for *in situ* hybridisation results of Runx2 and Ihh, which were derived from mutant KO mice, raised in our own colony at TU Darmstadt.

#### Quantification of hybridization signals and histological stainings with imageJ

Briefly, images were captured using bright field microscopy by ZEISS Axiophot microscope. Histological assessments of Alcian blue, Alizarin Red and Alkaline Phosphatase staining and IHC markers for Col-X, Ihh and MMP-13 were performed. For quantifying *in situ* hybridization signals, we used a purple-blue color from NBT formazan, which is a generated product of alkaline phosphatase reaction using BCIP and NTB substrate. In order not to interfere with the mRNA signal, we renounced on the counterstain. Before using ImageJ (freely available on http://rsb.info.nih.gov/ij/download.html.), the area, minimum and maximum grey area, and mean grey value can be selected in Set Measurements under Analyze. We used the tool “free hand selections” surrounding areas for measurements. All measurements were read clicking on Measure under Analyze. All areas measured for a hybridization signal were corrected for background reading. To define background, 5 areas without hybridization signal were selected and averaged, then subtracted from the signal value. All obtained numbers are percentages of stained area of the same correlating bone sections (n = 5 sections from 3 mice per group, and total 15 units were measured in each group), or of the entire micromass culture (n = 5 with separate replicas for each treatment and total 20 units were measured in each experimental setup). For Alcian blue, Alizarin Red and Alkaline Phosphatase staining, the minimum and maximum grey area and also the mean grey value were adapted to the corresponding colour.

### Microscopy and photography

Wholemount wild type and mutant mice, and also their upper and lower extremities stained with A-blu and/or A-red were analyzed and photographed using a ZEISS stereomicroscope using digital imaging with a TZ7 Panasonic camera. Microscopic images of immunostained cryosections were generally taken with bright field microscopy using a ZEISS Axiophot microscope. Using a ZEISS stereomicroscope, the three-dimensional micromass cultures were imaged and merged into a 2D stack.

## Results

### 1. Cholinesterase deletion mutants present strong phenotypes

**Fig 1:**
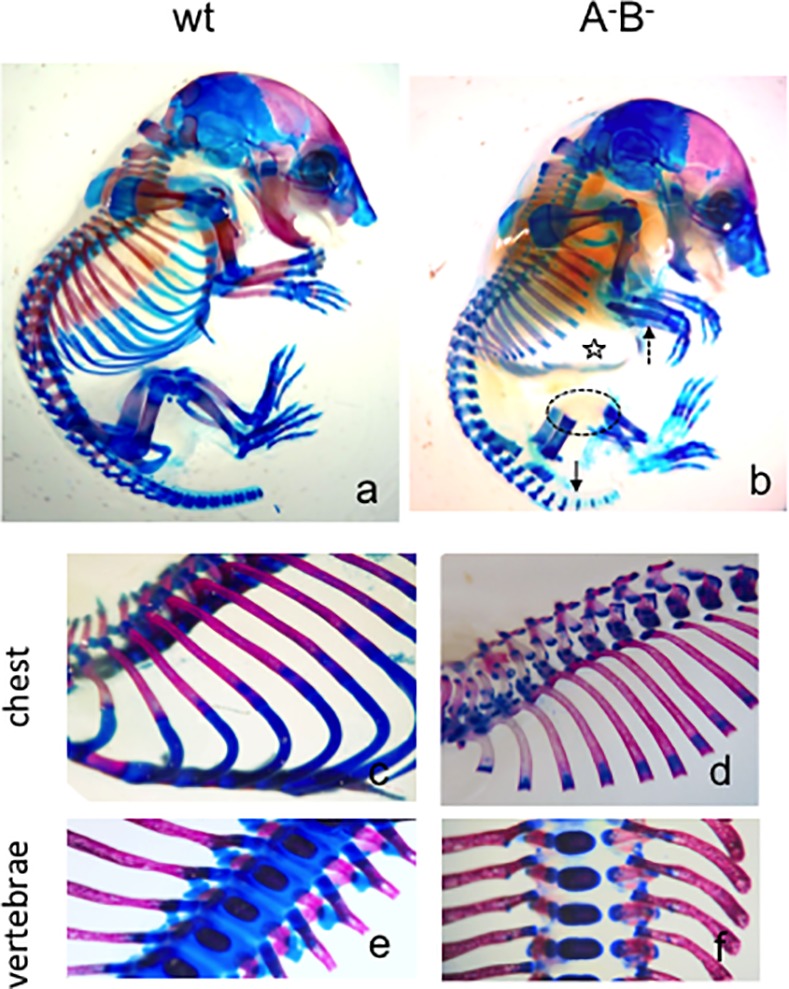
Newborn cholinesterase double-KO mice present strong skeletal phenotypes. Whole-mount staining by Alcian blue (A-blu) for cartilage (blue) and Alizarin red (A-red) for mineralizing bone elements (red) of P0 wild type (a) and A^-^B^-^ double-KO mouse (b). Note, in mutants A-blu staining is stronger in areas where mineralization is decreased, e.g. in distal long bones (b, stippled arrow) and dorsal ribs; A-blu is absent in ventral ribs (b, star) and hind limb joints (b, stippled circle). (c-f) details of mice in (a, b) of chest and vertebral column in wt (c, e) and A^-^B^-^ mutant (d, f). Further see text; see supplemental S1 Fig for A-blu staining of whole-mount mice only.

To elucidate the role of cholinesterases during early skeletogenesis *in vivo*, we characterized the phenotypes of AChE^-/-^/BChE^+/+^, AChE^+/+^/BChE^-/-^ and AChE^-/-^/BChE^-/-^ KO mice from E13.5 until birth (for simplicity, these are furtheron called A^-^B^+^, A^+^B^-^ and A^-^B^-^, respectively). Compared to wild type mice (wt), all three KO mice grew faster during their first two weeks of gestation, but slowed down in the last gestational week, as demonstrated by representative images, body weight and body length for A^-^B^-^ mutant (S1 and S2 Figs; [15]). While at E13.5 the weight of the A^-^B^-^ mutant was more than 3-fold more than that of wild type, by E16.5 its weight was 15–20% less than that of wt (S2 Fig). Furthermore, the mutant mice exhibited a strongly bended shape, a thickened skin, which was leathery rough (S1 Fig). In order to analyze skeletal structures of embryonic wild type and A^-^B^-^ double-KO mice, Alcian blue (A-blu) and Alizarin red (A-red) stainings were used to outline cartilage or mineralizing bone structures, respectively. To provide an overall picture of whole mice at birth, Fig 1 presents double-stained whole mount samples of a wild type (Fig 1A) and an A^-^B^-^ double-KO mouse (Fig 1B) at P0. For comparison, another pair of mice was stained only by A-blu (S3 Fig). Striking differences in endochondral ossification were observed, *in toto* documenting that skeletogenesis in mutant mice was severely affected. For example, in A^-^B^-^ mutant mice, A-red staining of their dorsal ribs was somewhat weaker, but extended farther ventrally (cf. Fig 1C and 1D). At the same time, mutant vertebrae and ventral ribs remained unstained by A-blu (cf. Fig 1A, 1B, 1E and 1F). The caudal vertebral columns in the mutants, A-blu staining was significantly diminished compared to wild type (wt). Several distinct differences of stainings in the skull were also noted. For example, head plates were stained more strongly by AB (however note: the skull will not be further discussed, since it follows the process of direct ossification).

#### Growth of long bones is accelerated in ChE mutants

**Fig 2:**
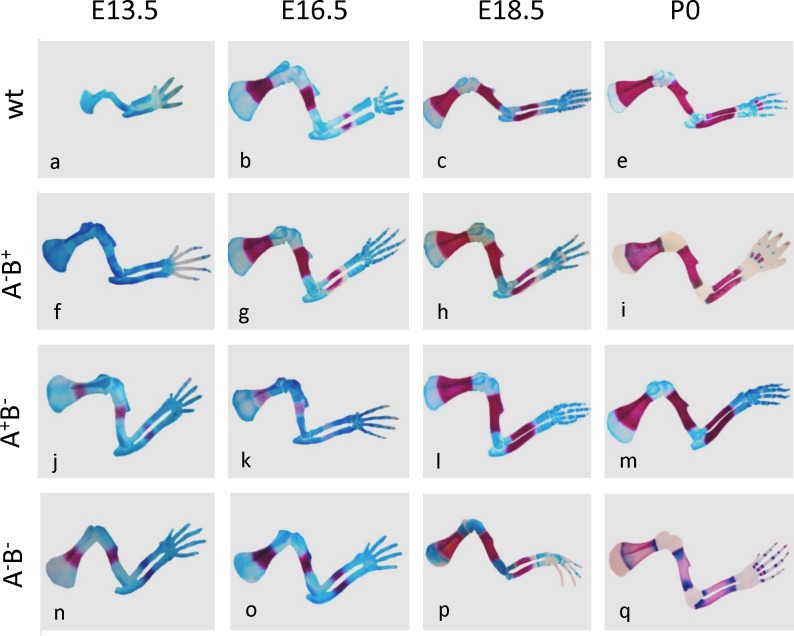
Cartilage matrix (blue) and mineralization (red) are changed in ChE mutants from E13.5 until birth. Note larger size of all mutants at E13.5 (cf. S1 and S2 Figs), and earlier onset of mineralization in A^+^B^-^ and in A^-^B^-^ mutant legs. Note earlier and stronger degradation of cartilage matrix (A-blu) in A^-^B^+^ and more so in A^-^B^-^ mutants, along with appearance of A-blu^+^ cells in their diaphyses. Size relations between legs are approximated.

To analyze cartilage and bone formation in one representative body part of all three mutants in detail, Fig 2 presents developmental series of A-blu and A-red double-stained in the front limbs from wild type and three mutant mice (Fig 2; with a temporal delay, hind limbs showed similar results). At E13.5, all three mutants had reached a much larger size as compared to WT. Notably, mutant limb development was approximately two days ahead of wild type (see also S1 and S2 Figs; cf. [15]). At this time, in mutants with deleted BChE (A^+^B^-^, A^-^B^-)^ mineralization had clearly commenced, while in wt and the A^-^B^+^ mutant A-red staining was still absent. As development proceeded, mineralization appeared rather normal. By P0 in A^-^B^+^, and more conspicuously so in A^-^B^-^ mutant (Fig 2I and 2Q), A-blu staining was absent in epiphyses, but now was found at each end of diaphyses, admixed with A-red staining. In summary, absence of either ChE had accelerated early limb growth, absence of BChE preferentially accelerated mineralization, while absence of AChE affected more strongly cartilage remodeling.

#### Growth plate and cartilage remodeling is disturbed in newborn ChE mutant mice

**Fig 3:**
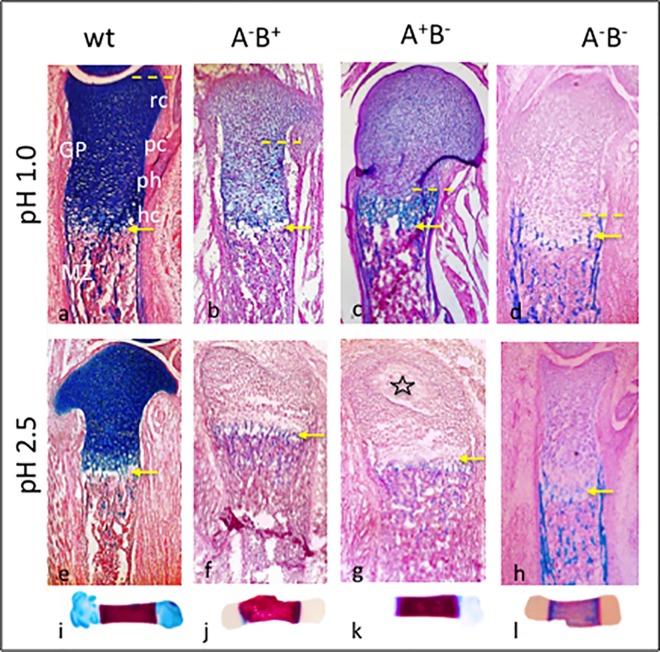
Cartilage remodeling is accelerated in perinatal ChE mutants. (a-h) Cryosections of WT and three ChE mutant femurs at P0 were stained by Alcian blue (A-blu) at pH 1.0 (upper; revealing sulfated proteo- and glucosaminoglycans, GAGs) and at pH 2.5 (lower; revealing carboxylated and weakly sulfated glycoproteins and mucopolysaccarides, PGs). Compare strong staining of epiphyses of WT with step-wise advanced degradation of A-blu in epiphyses of mutants (yellow stippled line and arrow), and its appearance in diaphyses. Note onset of secondary ossification in (g, star). (i-l) Whole-mounted femurs were double-stained by A-blue and A-red at pH2.5. rc, resting chondrocytes; GP, growth plate; MZ, mineralizing zone; pc, proliferating chondrocytes; ph, pre-hypertrophic chondrocytes; hc hypertrophic chondrocytes. Cryosections were counterstained by nuclear fast red. n = 5 per each sample. Further see text.

**Fig 4:**
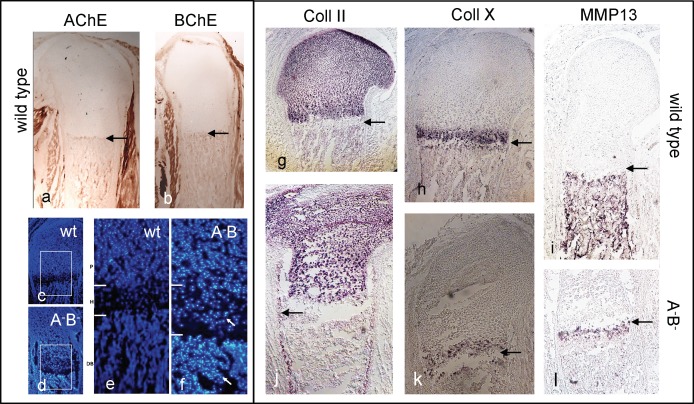
**(a, b) Expression of AChE and BChE in diaphysis of WT.** Note ChEs were also expressed on parts of perichondrium and periosteum, but were completely absent in epiphyses. **(c -l) Major matrix components and GP structure are changed in P0 A**^**-**^**B**^**-**^ **mutant mice.** (c-f) DAPI staining to detect specifically cell nuclei in GP of A^-^B^-^ KO and WT mice at P0 at low (c, d) and higher magnification (e, f). Note many brightly stained, mostly apoptotic cells in mutant. White arrows indicate high numbers of abnormally positioned of cells in mutant growth plate. (g-l) Serial longitudinal sections of distal femoral growth plates from newborn WT (g-i) and mutant hind limbs (j-l) were hybridized with Col-II (g, j), Col-X (h, k) and MMP-13 (i, l) riboprobes. Note that in mutant both Col-X and MMMP-13 were strongly reduced, while Col-II was still quite high in epiphysis and in periosteal cells on diaphysis of mutant mouse. n = 5 per each sample.

The epiphysis (growth plate, GP; Fig 3A; see also *Comments on terminology*) in a long bone of a newborn wild type mouse consists of the following areas: resting (rc), proliferating (pc), pre-hypertrophic (ph), and hypertrophic chondrocytes (hc). Below a narrow cell-free gap (called *degeneration zone* (DZ), or *chondro-osseous junction*, COJ; Fig 3A, arrow), the diaphysis with mineralizing bone follows (further-on called mineralizing zone, MZ; Fig 3A). In wt bones at P0, AChE or BChE activities were restricted to MZ and specific places of perichondria and periostea (Fig 4A and 4B); both ChE activities were completely absent in A^-^B^-^ mutant sections (not shown; see also [42, 44]). Notably, a sharp border restricted their expressions at the dia-/epiphysis border (arrows in Fig 4A and 4B). Consistently in all mutants the structure of growth plates, in particular of the degeneration zone (DZ) appeared widened, often even destructed (cf. Fig 4C–4F). DAPI staining of cell nuclei revealed many brightly stained, mostly apoptotic cells, spread over most of the epiphysis and reaching even into the mutant mineralizing zone (MZ, details in Fig 4F; further see Discussion); these were absent in wt mouse (Fig 4C and 4E).

Since cartilage remodeling in mutants appeared severely impaired, most prominently so between E18.5 and P0, we compared structural and molecular changes in femural cryosections of P0 mutants with wt bones (Fig 3). Sections were stained with Alcian blue (A-blu) at pH 1.0, revealing sulfated glucosaminoglycans (GAGs; Fig 3, upper), while A-blu staining at pH 2.5 visualized carboxylated and sulfated proteoglycans (PGs; Fig 3, lower), In wt mice, epiphyses were strongly stained at both pHs (Fig 3A and 3E), while in their diaphyses only some remnant A-blu staining was detected, which appeared somewhat stronger at pH 1.0 (Fig 3A). In all three ChE mutants, A-blu staining at both pH values was completely different from wt mouse. At pH 2.5, it was nearly absent in all three mutants (Fig 3F–3H and 3J–3L). However at pH 1.0, A-blu staining presented a differential picture. In A^-^B^+^ mutant, cells in epiphysis showed an increase in A-blu staining from resting (rc) to hypertrophic zones (hc), reflecting a decreasing loss of A-blu from rc to hc (Fig 3B). In A^+^B^-^ mutant A-blu was restricted to a narrow band of hypertrophic cells (hc), while staining in the mineralizing zone (MZ) and in parts of perichondrium was significant (Fig 3C). Finally in double A^-^B^-^ mutant, A-blu staining was nearly abolished in epiphysis, but now was most prominent within MZ (Fig 3D). Thus, absence of ChEs led to a premature (or, accelerated) degradation of cartilage matrix in epiphyses, whereby carboxylated and sulfated proteoglycans (PGs) were affected more significantly, earlier than sulfated glucosaminoglycans (GAGs). Most importantly, effects of both ChEs appeared additive in the A^-^B^-^ mutant. In summary, absence of either ChE selectively affected the ECM composition in epiphyses, documenting their strong accelerating effects on remodeling of cartilage.

#### Skeletal master genes are disturbed in growth plates of A^-^B^-^ mice

Collagen-II is a reliable marker of pre-hypertrophic chondrocytes, which is exchanged for collagen-X, as these cells become hypertrophic [19, 30]. As shown by ISH in wt mouse at P0, Coll-2 was found almost exclusively in the epiphysis, with increasing staining towards the hypertrophic zone (Fig 4G). In this zone of wt, Coll-10 was strongly expressed in a wide and a separate narrow band, from which few stained cells reached into the diaphysis (Fig 4H). MMP13 is a major protease involved in cartilage degradation, which was found in the mineralizing zone of wt mice (below DZ, arrow, Fig 4I). The distributions of all three markers were severely affected in A^-^B^-^ mutant (Fig 4J–4L; for simplicity, only A^-^B^-^ mutant is presented; results for the two others were comparable). At P0 Coll-2 was decreased in the epiphysis (Fig 4J; see further Discussion); Coll-10 was weakly expressed (Fig 4K). MMP13 was almost completely lost over the entire diaphysis; only some small band was left below DZ (Fig 4L). Together, these experiments revealed an extensive derangement of major cartilage constituents in the A^-^B^-^ mutant, and show that cartilage matrix degradation in the mutant diaphysis was disturbed (cf., Figs 2 and 3, S4 Fig).

**Fig 5:**
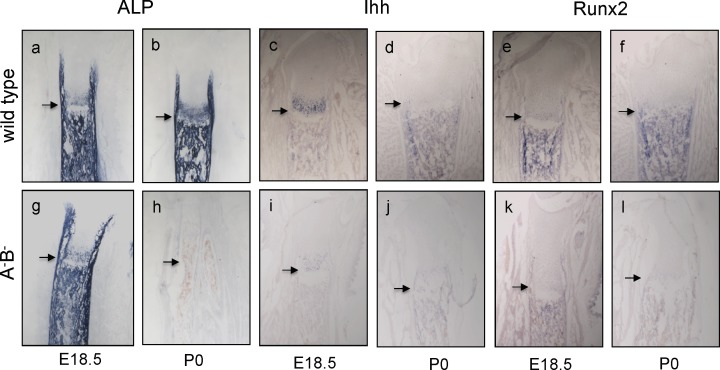
Changes of major gene expressions in A^-^B^-^ mutant tibias between E18.5 and P0. (a, b, g, h) ALP as expressed in hc and diaphysis of WT (a, b), disappeared completely by P0 in mutant (g, h). **(c-f, i-l) Expressions of Ihh and Runx2 disappear in mutant tibias.** In WT, ISH staining for Ihh expression shifted from a small zone in epiphysis into the diaphysis (c, d), while Runx2 was expressed at both stages in diaphyses (e, f). Note that in mutant at P0 both markers were not detectable anymore (j, l); however, in the E18.5 mutant, Ihh was nearly absent (i), while Runx2 was weakly expressed in diaphysis (k). n = 3 per each sample.

ALP, which marks hypertrophic cells before mineralization onset, is essential for normal mineralization [49–51]. The expression of this enzyme is lost in the mutant animals at P0 but comparable to wt by E18.5 (cf. Fig 5A and 5B with 5G and 5H; S4 Fig). The expression patterns for Ihh and Runx2, as analyzed by *in situ* hybridization (ISH) on long bones of E18.5 and P0 wild type mice, provided explanations for these changes (Fig 5C–5F and 5I–5L). *Ihh*, a marker of pre-hypertrophic chondrocytes and a regulator of Runx2, was strongly expressed in wt mice at E18.5 in a limited band of the epiphyseal HC zone. This pattern had almost completely shifted into the distal mineralizing zone by P0 (Fig 5C and 5D). Thus, *Ihh* shifted to positions where Runx2, a marker for hypertrophic chondrocytes, was expressed in wt mice at both time-points (Fig 5E and 5F), showing that in wild type between E18.5 and birth *Ihh* became co-localized with Runx2. In contrast in the mutant (Fig 5I–5L), both genes were expressed at a very low level at E18.5, whereby Runx2 was weakly expressed in the mineralizing zone (Fig 5K). Taken together, in P0 mutant legs the expression of decisive genes and proteins that direct hypertrophic differentiation and transition into mineralization had become suppressed. Loss of cholinesterase activities results in dramatic defects in chondrocyte maturation and matrix remodeling in the hypertrophic and in mineralizing zones, including increased levels of apoptotic cells in the degenerating zone (DZ; Fig 4C–4F, S4 Fig).

### 2. Cholinergic regulation of skeletogenesis in mesenchymal chick limb micromass cultures

**Fig 6:**
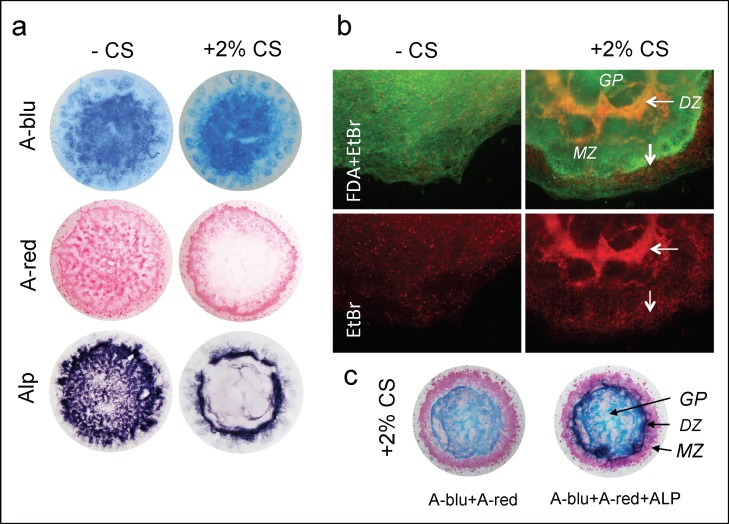
Micromass cultures from chicken limb buds as skeletogenic *in vitro* models: chicken serum reorganizes improves their spatial order. (a) Micromass cultures of chicken limb bud cells were incubated in absence (left) and presence (right) of 2% chicken serum (CS) for 13 days. Addition of CS leads to a stepwise chondrogenic differentiation, whereby cartilage forms in an inner tissue core (upper; equiv. to a *GP-like* area), followed by a ring of ALP (lower; equiv. to a *DZ-like* area) and mineralization (middle; equiv. to a *MZ-like* area) near the periphery, as shown by A-blu, A-red and Alkaline phosphatase (ALP) stainings. (b) Combined FDA/EtBr staining to detect living cells (green) and dead cells (red). (c) Combined A-blu/A-red staining (left), and merged with ALP staining (right). Zone of A-blu indicates proliferative and matured chondrocytes; zone of high cell death and ALP activity without proteoglycan matrix secretion marks the zone of hypertrophic and “terminal” hypertrophic chondrocytes. A-red staining identified calcification and marks the zone of chondrocyte-derived osteoprogenitor cells. n = 5 per each series. *DZ*, *in vitro* zone corresponding to DZ; *GP*, *iv* zone corresp. to GP; *MZ*, *iv* zone corresp. to MZ.

Based on the above findings with ChE mutant mice, and also on previous studies by Kawakita et al. [25], we hypothesized that the defects on epiphyseal growth plate chondrocytes were mediated through neuronal nicotinic acetylcholine receptors (nACHRs). To test our hypothesis *in vitro*, we utilized micromass cultures of primary embryonic limb bud mesenchyme [46]. In this regard, mesenchymal progenitor cells undergo condensation into compact aggregates (so-called *nodules*), prior to differentiation into chondrocytes and synthesis of cartilage-specific ECM components, such as collagens and proteoglycans (review, [52]). However, using the conventional technique it was not possible to distinguish the spatial arrangement of resting, proliferative and hypertrophic chondrocytes (cf. Fig 6A, left,—CS). Based on our culturing experiences with retinal spheroid cultures [15, 53], we investigated whether supplementation with 2% chicken serum (CS) could improve the spatial organization in mesenchymal micromass cultures. Under conventional culture conditions, we observed typical cellular condensation, differentiation and extracellular matrix secretion (Fig 6A, left). In contrast, with supplementation with 2% chicken serum (Fig 6A, right) a distinct separation of cartilage matrix (stained by A-blu), calcified matrix (A-red) and differentiated chondrocytes (ALP staining) was observed, whereby the differentiation process spread from inside to the periphery (Fig 6A and 6C; +2% CS). To analyze the effect of chicken serum on cell viability, we used FDA and EtBr stainings (Fig 6B). In absence of CS, a low number of dying cells was distributed evenly in micromass cultures, whereas the addition of CS resulted in a circular arrangement of areas with high cell death (EtBr, orange/red in Fig 6B). This ring corresponded with a ring of ALP activity (cf. Fig 6A and 6C), the latter marking terminal hypertrophic chondrocytes. These results indicate that chick limb micromass cultures supplemented with 2% chicken serum differentiate circularly from inside out, thereby following distinct differentiation steps of chondrogenesis (Fig 6C).

**Fig 7:**
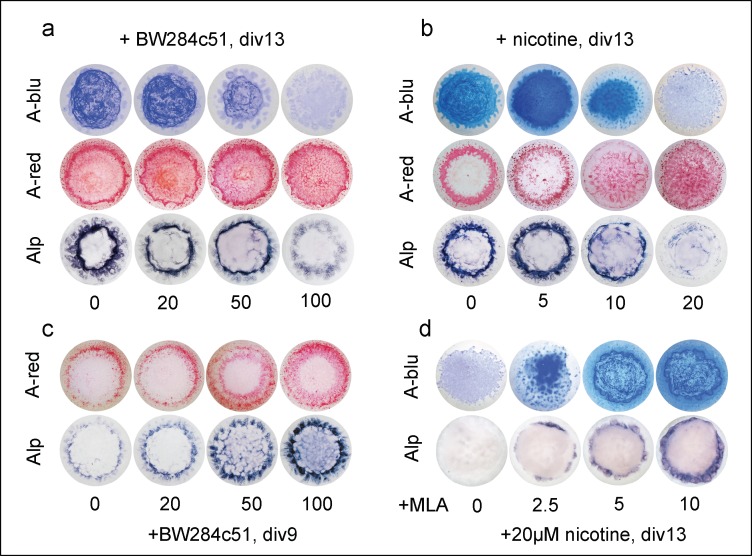
AChE inhibition or stimulation of nAChRs reduces proteoglycan content and ALP activity, and increases mineralization *in vitro*. (a-d) Micromass cultures of chicken limb-bud cells of HH stage 22–24 were stained with A-blu, A-red and for ALP activity. Mesenchymal micromass cultures were incubated for 13 days (a) or 9 days (c) in presence of increasing doses of BW284c51, or control conditioned medium. (b) Chick limb micromass cultures were incubated for 13 days in the presence of increased doses of nicotine or control conditioned medium. (d) Combined cultivation of chick limb micromass cultures with 20μM nicotine plus increasing doses of MLA. ALP staining of div9 micromass cultures (c) indicated dose-dependent premature chondrogenesis at early stages leading to a suppressed chondrogenic differentiation at later cultivating times (a). All concentrations are indicated as μM; n = 7 per each treatment.

Using this improved culture procedure, we investigated cholinergic regulation of chondrogenesis and cartilage matrix secretion (Fig 7). At div 9, a time when only mineralization sets in, the AChE inhibitor BW284c51 increased calcification and ALP activity in a concentration-dependent manner (Fig 7C, AB staining is not affected by BW284c51 at this stage, not shown). By div 13, BW284c51 led to a concentration-dependent decrease of A-blu staining, regressing from periphery to the center (Fig 7A). In parallel, calcification expanded towards inside, and ALP activity disappeared (Fig 7A, A-red and ALP). To analyse an involvement of nAChRs, micromass cultures were treated with nicotine, an agonist of ACh (Fig 7B). At a low concentration of 5μM nicotine, A-blu staining was increased, indicating an advanced initiation of pre-chondrogenic condensation and fusion of nodules of chondrocytes (Fig 7B). At higher nicotine concentrations A-blu staining (i.e., proteoglycan contents) decreased in a concentration-dependent manner, reaching very low levels at 20μM of nicotine. Similarly, ALP activities also decreased, being nearly absent at the highest nicotine level. In contrast, calcification increased simultaneously and reached into the inner cell mass of the micromass cultures at div13 (cf. quantification in S5 Fig). Thus, not only inhibition of AChE, but also cholinergic stimulation of nAChRs promoted cartilage-characteristic matrix secretion and “terminal” hypertrophic differentiation of chondrocytes. Then, we applied 20μM of nicotine together with increasing concentrations of MLA, a specific inhibitor of the α7-nAChR (Fig 7D). In absence of MLA (control), both A-blu and ALP stainings were very weak (cf., Fig 7B and 7D; S5C Fig), while with increasing concentrations of MLA both A-blu and ALP stainings were completely restored in a concentration-dependent manner (Fig 7D). Thus, terminal hypertrophic chondrocyte differentiation was mediated *in vitro* at least in part through cholinergic stimulation via the α7-nAChR.

**Fig 8:**
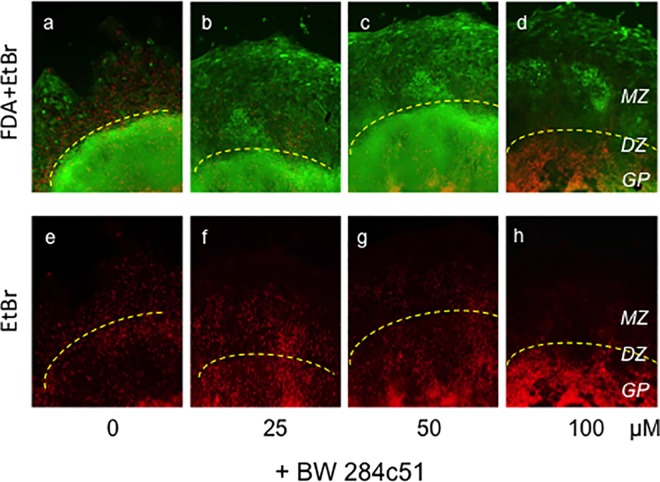
Inhibition of AChE accelerates progression into cell death in chondrogenic zone, as shown by a FDA/EtBr dual staining of micromass cultures on div13. Cell viability and cell death were revealed by combined FDA/EtBr staining for living and dead cells (green, red, respectively). Note with increasing BW284c51 concentrations dead cells are increased in inner compartment of micromass culture, corresponding with chondrogenic zone of GP (*GP*; below stippled lines), but decrease in outer compartment, corresponding with MZ. n = 3 per each sample. *DZ*, *in vitro* zone corresp. to DZ; *GP*, *iv* zone corresp. to GP; *MZ*, *iv* zone corresp. to MZ.

As further support for a stimulatory role of inhibited AChE in promoting terminal chondrogenic differentiation *in vitro*, we used a combined FDA/EtBr staining (live cells are green, dead cells are red), which allowed an easy distinction of *in vitro* zones, corresponding *in vivo* with chondrogenic (*GP*), degenerating (*DZ*) and mineralizing zones (*MZ*; cf. Figs 6 and 8 with 3). Application of BW284c51 showed that cell death in the inner core of div13 micromass cultures strongly increased in a concentration-dependent manner (red, Fig 8; *GP*, below stippled lines), while it decreased in their outer compartments. Concomitantly, cell viability (FDA, green) decreased in chondrogenic, and increased in mineralizing zones. These findings again demonstrate that inhibition of AChE activity accelerated hypertrophic differentiation and their death, while proliferation (by viability test) increased, indicating a decisive involvement of AChE on chondrocytes in epiphyses (growth plates).

## Discussion

Here we have analyzed three mutant embryonic mice, in which AChE, BChE or both genes were deleted [42–44]. All three mutant mice presented strong, yet distinct skeletal phenotypes. Our *in vivo* findings in mice were supported by an improved *in vitro* 3D micromass culture model, using mesenchymal stem cells from chicken embryonic limbs [46] in which not only AChE inhibition, but also nicotine promoted *in vitro* bone development. The present study extends a recent *in vivo* study on living chicken embryos, in which beads soaked with cholinergic components were implanted into one limb bud ([13], see below).

### Acetylcholine functions in epiphyses of forming bones

**Fig 9:**
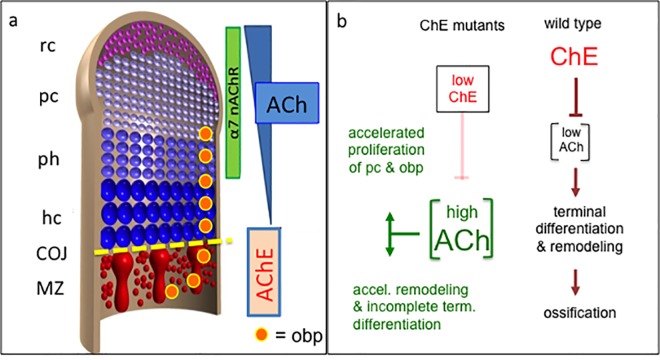
Models of cholinergic balancing of chondro- vs. osteogenesis. (a, left) scheme of the growth plate of developing long bones, including four different states of chondrocytes (rc, pc, ph, hc), before cells reach the mineralizing zone (MZ, red). Epiphyseal and diaphyseal spaces are separated by a chondro-osseous junction (COJ; also called *degeneration zone*, DZ). Note that osteoblastic precursors (obp, orange circles), a second source of proliferative cells besides pc, will migrate from GP into MZ [31]. (a, right) represents spatial distributions of ACh, of α7-nAChR and of AChE. Note that in regions of ChE activity in MZ, ACh concentration and thus, cholinergic signaling will be low (cf. Fig 4A and 4B). (b) depicts two effective pathways of AChE actions. (left in b) whenever systemic AChE is low (e.g. in ChE mutants), systemic ACh will be high, leading to increased/advanced proliferation of both proliferative chondrocytes (pc) and osteoblastic precursors (obp). (b, right) At normal levels of ChEs, as in diaphyses of wt, a lower level of ACh in GP will allow chondrocytes to normal terminal differentiation, remodeling and mineralization. Further see Discussion. rc, resting chondrocytes; pc, proliferating chondrocytes; ph, pre-hypertrophic chondrocytes; hc, hypertrophic chondrocytes; obp, osteopblastic precursors; COJ, chondro-osseous junction; MZ, mineralizing zone.

The main synthesizing enzyme for ACh is ChAT, with a minor possible contribution by carnithin acetyltransferase (carAT, [5]). Stem cells in the resting zone of growth plates produced ACh [5, 32], as do cultured osteoblasts [4, 27]. In vivo, ChAT has been identified in early mesenchyme and chondroblasts of the chick limb bud [8, 25, 26]. Specific types of AChRs have been detected in resting, proliferating and pre-hypertrophic chondrocytes (incl. α7-nAChR) of the murine epiphysis (growth plate, GP), as well as in osteoblasts, spongy bone and periosteum, but not in hypertrophic chondrocytes ([4, 25, 26]; see schematic, Fig 9A). Muscarinic and nicotinic types of AChRs were found in osteoblasts *in vitro*, and have been shown to be involved in their proliferation and differentiation [27]. *In vivo*, a major fraction of osteoblasts originate from the epiphysis, migrating into the diaphysis (cf., string of highly Coll-2^+^ cells; arrow in Fig 4J; [32, 54–58]). Therefore, ACh and the responding elements of cholinergic stimulation are preferentially distributed in epiphyses of forming vertebrate long bones, there increasing proliferation of *both* chondroblasts and osteoblasts (schematic, Fig 9, see further below).

### Cholinesterases are rare in epiphyses of forming bones

In considering how absence of ChEs could affect bone development, their distinct *in vivo* localization needs attention. Besides their expression along perichondrium and periosteum, both AChE and BChE were found in diaphyses of the wild type long bones (Fig 4A and 4B). Of note, AChE is expressed in cultured osteoblasts [11, 59]. ChE activities were absent inside the epiphysis, except a narrow band of AChE^+^ cells in/near the degeneration zone (DZ, see below). Although ChEs are probably secreted by osteoblasts [11, 12, 60], significant amounts of both ChEs [61] are most likely delivered to the diaphysis via the blood supply. This process itself is regulated by cholinergic mechanisms [62–64], a topic which is not considered here. Immediately after vascularization, mineralization commences. This suggests that areas of ACh action and of its degradation are locally separated in epiphysis and diaphysis, respectively (cf. Fig 9). Hence, cholinesterases in diaphyses could act as sinks for ACh to effectively degrade it, since ACh as a very small molecule can easily diffuse from epi- into diaphysis (schematic, Fig 9A). Therefore it appears likely that in mutant mice in which either one, or both ChEs were missing, levels of ACh in epiphyses of forming bones were elevated.

### Lack of cholinesterases disrupts orderly matrix remodeling

Regulators of endochondral ossification [21–24, 65–69], which were disrupted in ChE mutant mice, act predominantly in the epiphysis. The Runx2 protein, a master regulator of chondrogenesis [19–22, 30, 65], has a binding site on the AChE promotor [18], which could be relevant for AChE expression near the degeneration zone. In ChE mutants the period between E18.5 and birth showed an extremely rapid remodeling of the cartilage matrix, that is epiphyses became void of A-blu and ALP stainings (see Figs 3–5). As a consequence of absence of Ihh and Runx2 expressions in the newborn A^-^B^-^ mutant, also MMP-13, Col-X and ALP were also nearly absent (cf., S4 Fig). In micromass cultures, the effects of AChE inhibition on ALP were perplexing at a first glimpse (Fig 7). At div 9, when remodeling was still ongoing, increasing AChE inhibition drove the system towards mineralization (note in Fig 7C: ALP also in inner core of micromass). However by div 13, mineralization became restricted to the micromass periphery, indicating its near completion (now, inner core was free of ALP; Fig 7A). Hence at div 13, ALP expression decayed, which was again promoted by increasing AChE inhibition.

### Does elevated ACh in epiphysis stimulate chondroblast proliferation?

The observations presented here, using completely different approaches, are consistent with the notion that bone development is accelerated by an underlying nicotinic stimulation. However, it has been reported that nicotinic stimulation could extend chondrogenesis [5]. This is further strengthened by the observation that maternal nicotine exposure resulted in somewhat smaller sizes of α7-nAChR^+/+^ fetuses by E15.5, but not in α7-nAChR^-/-^ fetuses [25]. The apparently conflicting results compared to our study may be due to the fact that effects of nicotine in different systems are difficult to compare. For instance, nicotine affected osteosarcoma cells dose-dependently. That is, their proliferation was *stimulated* at lower concentrations, but was *inhibited* at higher doses [33]. More specifically, the growth curves of ChE mutant embryos document (S2 Fig), that their sizes and weights were much advanced by E13.5, and were accompanied by acceleration of both chondrogenesis and mineralization (Fig 2). But after E13.5, their growth rates slowed down (S1 and S2 Figs), so that from E16.5 onwards ChE mutant mice remained smaller until birth. Thus, a larger population of chondroblasts needed longer time to go through all steps of chondrogenesis, such that an *extension* brought with it a *delay*, which compromised an orderly matrix remodeling (Fig 9B, left pathway). For instance, cells with matrix features of pre-hypertrophic chondrocytes (e.g., A-blu^+^ staining) found themselves in the mineralizing zone (Figs 2 and 3; see further below), showing that a) these cells had not completed matrix remodeling before their arrival in the diaphysis, and that b) the process of ossification in ChE mutants was initiated *prematurely* and *independently* of completion of chondrogenesis. Taken together, our observations are consistent with the notion that nicotinic stimulation primarily regulates the size of the chondrocyte population [25].

### Each cholinesterase exerts distinct skeletogenic effects

Since all three phenotypes presented distinct differences, further specific contributions from either cholinesterase have to be considered. BChE has been associated with sustaining cell proliferation in development and cancers [7, 10, 14]. In most cases, including bone development, BChE may exert its effects by degrading ACh. Its availability in bones depends—at least partially—on blood supply (see above). Noticeably, supplementation of micromass cultures with chicken serum (CS) provided them with higher levels of BChE [70]. Indeed, BChE in CS could have promoted their spatial restructuring (Fig 5), since BChE had improved a laminar structure in retinal spheroids [53]. The accelerating effect of BChE absence was most obvious in the epiphysis of the A^+^B^-^ mutant, where the process of secondary ossification had clearly commenced at birth (Fig 3G, star). In wild type mouse, this does not happen before P6/7 [71], indicating that development of long bones in this mutant mouse was advanced by almost one week! A-blu staining at pH1 (indicating sulfated GAGs) also demonstrated a massive acceleration of bone differentiation in absence of BChE (Fig 3C and 3D). In addition to elevating ACh levels, BChE could act indirectly by its complexing in blood with other proteins, e.g. transferrin [70], and thereby affect ALP and mineralization [72, 73].

Also for AChE additional actions must be envisaged, since in A^-^B^+^ embryos onset of mineralization was delayed after E13.5 (Fig 2), contrasting the other two mutant mice. This assumption is supported by bead implantations into chicken limb buds, whereby AChE inhibitors did not stimulate, but rather decelerated skeletogenesis [13]. In fact, AChE has been associated with several non-conventional activities, independent of its ACh-degrading capacity [7, 10, 15–17]. These include its actions on cell differentiation, on neurite outgrowth, in stress responses and on apoptosis ([74–77]; see Introduction). Cell adhesive mechanisms in epiphyses will organize cells during cartilage remodeling. Thus, specific surface features of AChE protein, such as their CHED domain [78], its peripheral anionic binding site (PAS, [14]), or its abilty to complex with other proteins, such as collagens, could be involved in separating cartilage matrix (A-blu) and ALP from MZ (A-red, Figs 2–4 and 6B). A tetrameric ColQ-form of AChE possesses a collagen tail [7, 14], which in turn can bind to ALP [79], the latter disappearing at onset of calcification [72] (cf., Fig 6A and 6C). Furthermore, an enzymatic side activity of AChE could also affect bone formation ([13, 80, 81]). In particular, AChE´s possible role in apoptotic processes is being much disputed [15, 74–77]. In growth plates of wild type mice, apoptosis occurs in a narrow stripe of cells near the epi-/diaphysis border, where a slight elevation of AChE activity was noted (not shown). Interestingly, in a zone of micromass cultures that corresponds precisely with this border (DZ; Fig 6B), cell death was strongly increased as AChE was inhibited (Fig 8E–8H). The precise molecular mechanism of how AChE affects apoptosis remains elusive, but could be due to AChE affecting nuclear condensation and caspase-9 activation [82].

## Conclusion

In conclusion, in cholinesterase knockout mice skeletogenesis was much disturbed, due to accelerated chondrocyte proliferation and premature onset of mineralization, leading to a premature expression of skeletogenic master genes and disturbed cartilage matrix remodeling. A primary effect of inhibition (or absence) of cholinesterase(s) appears to be an increase of chondroblasts, due to an elevated level of ACh. This conclusion is supported by *in vitro* micromass cultures from chicken limb buds. Thus, cholinesterases are major players to define local (graded) concentrations of ACh in the forming bone, which regulates periods of proliferation and differentiation of chondrocytes and of osteoblasts. Additionally, distinct roles of both ChEs most likely also contribute to skeletogenesis. These findings are biomedically highly relevant (see, Introduction and [33–40, 58]) and deserve much more investigations.

**Comments on terminology:** In the literature, different terms are used for the same components of growing bones. Therefore, our definitions follow here (cf., Fig 3A): the bone can be subdivided into *epiphysis* and *diaphysis* (*mineralizing zone*, MZ). In the developing bone, epiphysis is coincident with the *growth plate* (GP; also called *physis*); GP consists of *resting chondrocytes* (rc), *proliferating chondrocytes* (pc), *pre-hypertrophic chondrocytes* (pc), and *hypertrophic chondrocytes* (hc), followed by the *ossification fissure* (also called *degeneration zone*, DZ, or, *chondro-osseous junction*, COJ), and then MZ. However, at a more mature stage, a secondary ossification center will develop within the epiphysis (cf. Fig 3G); from now onwards epiphysis and GP are not congruent any longer.

## Supporting Information

S1 Fig General growth is accelerated in ChE mutant mice. (a) Huge size advance of A^-^B^-^ mutant fetus at E13.5; (b) representative photographs of A^-^B^-^ mutant and WT mice from E13.5 until P0. Note figure shows relative sizes of fetuses acc. to S2 Fig.
(TIFF)

S2 Fig **Quantification of body weight (a) and body length (b) of A**^**-**^**B**^**-**^ **mutant fetus mice and wt mice.** Mean values ± SEM are presented and sample numbers ("n") are indicated above bars.
(TIF)

S3 Fig Cartilage formation in A^-^B^-^ newborn KO mice is disturbed. Whole-mount staining by Alcian blue (A-blu) for cartilage (blue) of P0 wild type (a) and cholinesterase double-KO mouse (b). Note absence of A-blu in ventral ribs (star), or in joint regions (stippled circle).
(TIFF)

S4 Fig Quantification of ISHs from Figs 4 and 5 at E18.5 (upper) and P0 (lower), as determined by ImageJ analyses (see Materials and methods). Further see text.
(TIF)

S5 Fig Quantification of A-blu (AB), A-red (AR) and ALP stainings of micromass cultures from Fig 7A, 7B and 7D, as determined by ImageJ analyses (see Materials and methods). Further see text.
(TIF)

## Abbreviations

A-blu: Alcian blue
ACh: acetylcholine
AChE: acetylcholinesterase
ALP: alkaline phosphatase
A-red: Alizarin red
BChE: butyrylcholinesterase
BW284c51: 1,5-bis-(4-allyldimethylammoniumphenyl)pentan-3-one dibromide, a specific inhibitor of AChE
ChAT: choline acetyltransferase
Coll-II: collagen type II
GAGs: glycosaminoglycans
Ihh: Indian hedgehog
iso-OMPA: tetra-isopropyl-pyrophosphoramide, a specific inhibitor of BChE
MLA: methyllycaconitine, a specific inhibitor of α7-nAChR
MMP13: metalloprotease-13 (also called collagen-3)
nAChR: nicotinic acetylcholine receptor
PG: proteoglycan
Runx2: runt-related transcriptional factor-2
