# Supplementary figures and images for: Endochondral Ossification Is Accelerated in Cholinesterase-Deficient Mice and in Avian Mesenchymal Micromass Cultures
Source: PLoS One. 2017 Jan 24;12(1):e0170252. doi: 10.1371/journal.pone.0170252 (PMC5261733; doi:10.1371/journal.pone.0170252)

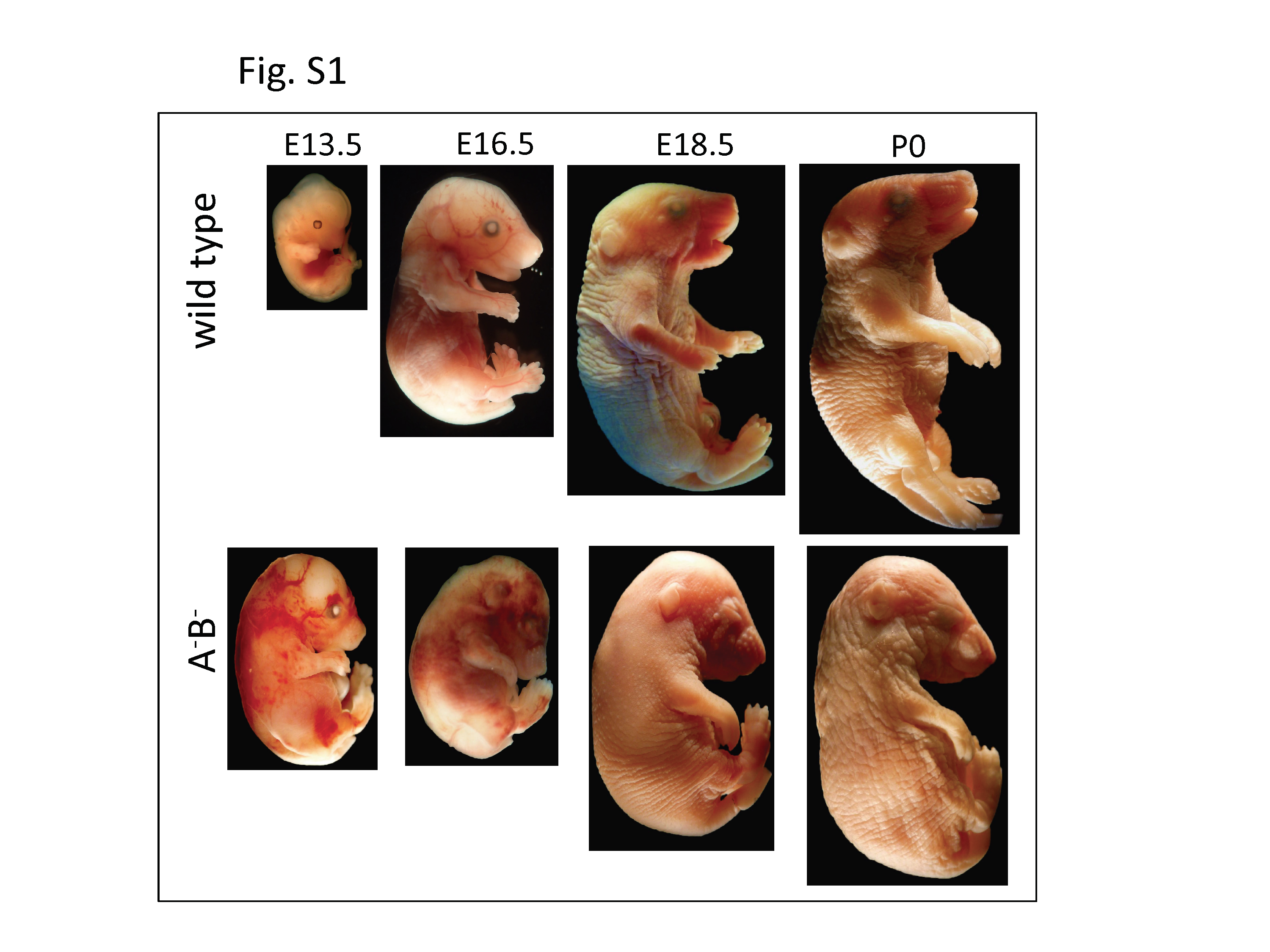

Supplement: S1 Fig — (a) Huge size advance of A-B- mutant fetus at E13.5; (b) representative photographs of A-B- mutant and WT mice from E13.5 until P0. Note figure shows relative sizes of fetuses acc. to S2 Fig. (TIFF) [file pone.0170252.s001.tiff]

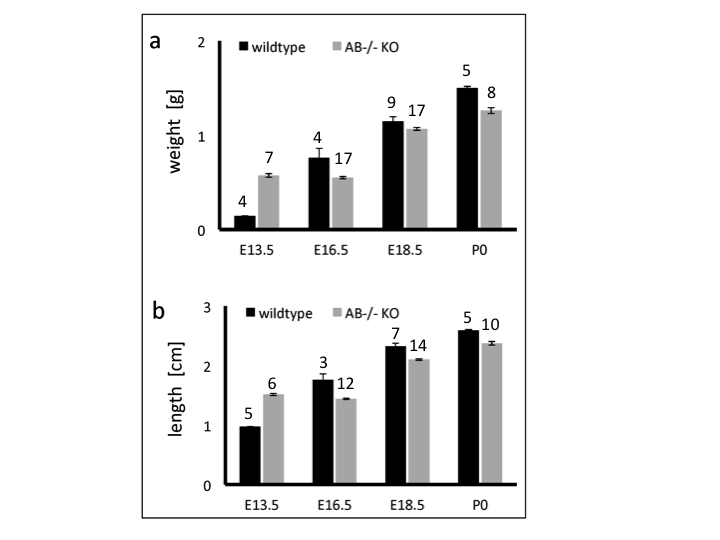

Supplement: S2 Fig — Quantification of body weight (a) and body length (b) of A-B- mutant fetus mice and wt mice. Mean values ± SEM are presented and sample numbers ("n") are indicated above bars. (TIF) [file pone.0170252.s002.tif]

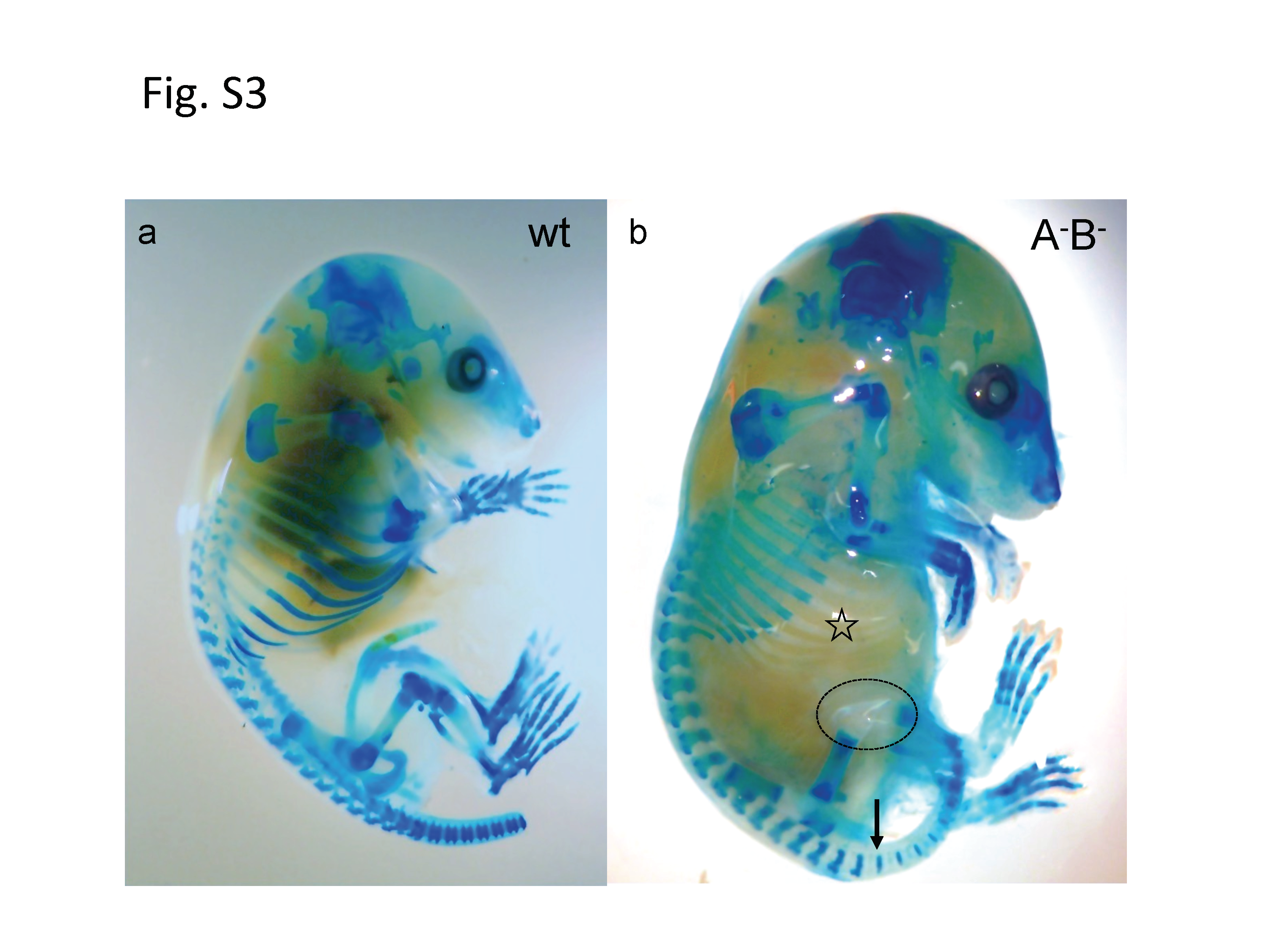

Supplement: S3 Fig — Whole-mount staining by Alcian blue (A-blu) for cartilage (blue) of P0 wild type (a) and cholinesterase double-KO mouse (b). Note absence of A-blu in ventral ribs (star), or in joint regions (stippled circle). (TIFF) [file pone.0170252.s003.tiff]

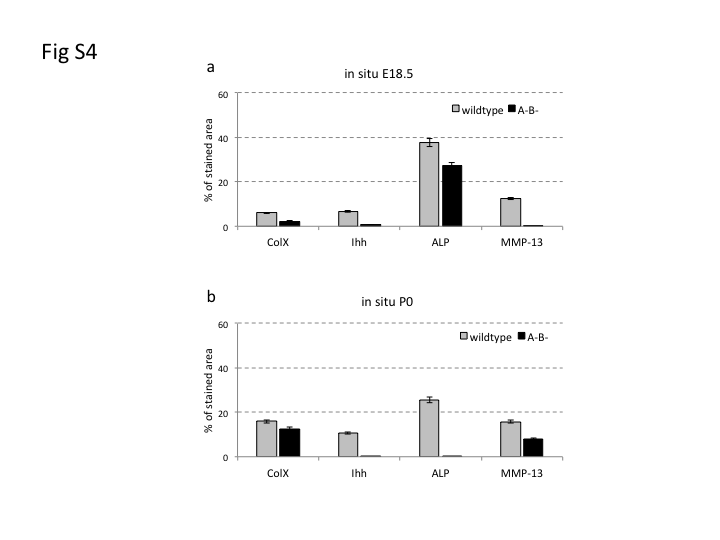

Supplement: S4 Fig — Further see text. (TIF) [file pone.0170252.s004.tif]

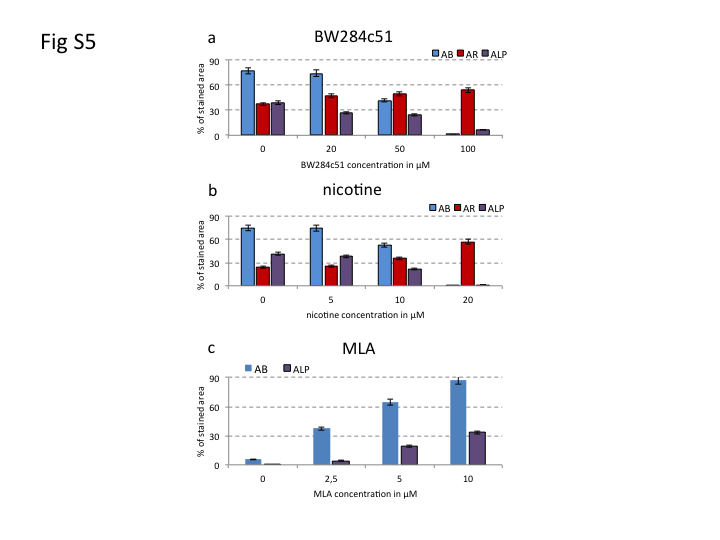

Supplement: S5 Fig — Further see text. (TIF) [file pone.0170252.s005.tif]
